# Architecture of the Bacterial Flagellar Distal Rod and Hook of *Salmonella*

**DOI:** 10.3390/biom9070260

**Published:** 2019-07-07

**Authors:** Yumiko Saijo-Hamano, Hideyuki Matsunami, Keiichi Namba, Katsumi Imada

**Affiliations:** 1 Graduate School of Frontier Biosciences, Osaka University, 1-3 Yamadaoka, Suita, Osaka 565-0871, Japan; 2 Molecular Cryo-Electron Microscopy Unit, Okinawa Institute of Science and Technology Graduate University, 1919-1 Tancha, Onna, Kunigami 904-0495, Japan; 3 RIKEN Center for Biosystems Dynamics Research and SPring-8 Center, 1-3 Yamadoaka, Suita, Osaka 565-0871, Japan; 4 Department of Macromolecular Science, Graduate School of Science, Osaka University, 1-1 Machikaneyama, Toyonaka, Osaka 560-0043, Japan

**Keywords:** bacterial flagellum, crystal structure, electron cryomicroscopy, flagellar rod, hook

## Abstract

The bacterial flagellum is a large molecular complex composed of thousands of protein subunits for motility. The filamentous part of the flagellum, which is called the axial structure, consists of the filament, the hook, and the rods, with other minor components—the cap protein and the hook associated proteins. They share a common basic architecture of subunit arrangement, but each part shows quite distinct mechanical properties to achieve its specific function. The distal rod and the hook are helical assemblies of a single protein, FlgG and FlgE, respectively. They show a significant sequence similarity but have distinct mechanical characteristics. The rod is a rigid, straight cylinder, whereas the hook is a curved tube with high bending flexibility. Here, we report a structural model of the rod constructed by using the crystal structure of a core fragment of FlgG with a density map obtained previously by electron cryomicroscopy. Our structural model suggests that a segment called L-stretch plays a key role in achieving the distinct mechanical properties of the rod using a structurally similar component protein to that of the hook.

## 1. Introduction

Many motile bacteria move in liquid environments by rotating a helical filamentous organelle called the flagellum. The flagellum is a large molecular assembly of about 20–30 thousand of protein subunits of more than 20 types of proteins. The filamentous part of the flagellum, termed the axial structure, is rotated by a motor embedded in the cell membrane. The axial structure consists of three morphologically distinct regions, the rod, the hook, and the filament from the proximal to the distal end, with a few minor components [1,2]. The filament is a long helical propeller with a diameter of ~20 nm and a typical length of around 15 μm. The filament is a helical assembly of about 30,000 flagellin (FliC) subunits. The hook is a short, curved segment with an approximate length of 55 nm. The hook is a helical assembly of about 120 subunits of FlgE [3,4]. The function of the hook is a universal joint that transmits motor torque to the filament in any orientation relative to the motor axis. The rod is a straight structure with a length of about 30 nm. The rod is a helical cylinder that comprises four proteins: FlgB, FlgC, and FlgF in the proximal part and FlgG in the distal part [5,6,7]. The rod is a drive shaft that penetrates the peptidoglycan (PG) layer and the outer membrane and connects the hook with the rotor of the motor.

The axial components share a common architecture in the subunit arrangement and the domain arrangement. The axial subunit proteins are arranged in a helical array of 11 subunits in two turns of the 1-start helix. This arrangement produces 11 protofilaments, which are strands of the component proteins aligned nearly parallel to the filament axis [8,9,10]. The subunits form a concentric multi-layer tube in the axial structure. The inner tube is composed of α-helical coiled coils constructed by the N- and C- terminal regions of each component protein [11,12,13,14,15]. These terminal regions are disordered in monomeric state in solution but are folded into the coiled coils only when the subunits are incorporated into the flagellum [16,17].

Despite the common architecture, the mechanical properties of the axial structures are quite distinct. The filament forms a stiff, super helical structure as a propeller for efficient propulsion of the cell. The hook is flexible in bending but rigid against twisting to achieve a universal joint function, whereas the rod is a rigid straight cylinder to work as a drive shaft. Since the inner most tube shows a common structural feature, the differences in the mechanical and functional properties are ascribed to the structural difference of the outer regions. In fact, the amino acid sequence of the outer region of FliC (UniProtKB ID: P06179) differs completely from that of FlgE (UniProtKB ID: P0A1J1) or FlgG (UniProtKB ID: P0A1J3). However, FlgE and FlgG show a high sequence similarity to each other, even in the first outer region D1, although FlgE has an additional domain outside D1. Therefore, their structures have been investigated to understand the molecular basis of the specific properties of these structures.

The hook structure of the *Salmonella typhimurium* (St) has been studied by X-ray crystallography and electron cryomicroscopy (cryoEM) image analysis. St-FlgE is composed of three domains, D0, D1, and D2, and a region connecting D0 and D1 termed Dc. The D1 and D2 domains are composed of β-structures and are connected by a short stretch of an anti-parallel β-strands [18]. The D1 and D2 domains are loosely packed along the protofilament in the hook, which allows the sliding motion in the axial subunit interface. Therefore, the intersubunit distance can be compressed or extended up to ~2 nm. This property is thought to be a key factor for flexibility in bending [18,19,20]. On the other hand, the D2 domains are closely arranged along the 6-start direction on the outer surface of the hook. This structure greatly contributes to rigidity against twisting [18,21]. However, the structure of the Dc region is unclear because of the low resolution of the cryoEM density. Recently, the complete hook structure of *Campylobacter jejuni* (Cj) revealed that D0 and D1 domains are linked by an l-shaped extended structure termed l-stretch, which is composed of 50 residues following the N-terminal helix [15]. The l-stretch of the Cj-hook interacts with the neighboring three protofilaments, thereby stabilizing the hook structure [15]. Therefore, the Dc region of St-hook would be expected to adopt a structure similar to the l-stretch of Cj-hook.

A partial structural model of the distal rod has recently been constructed on the basis of a cryoEM density map of the distal rod at a 7 Å resolution obtained from a polyrod mutant of St-FlgG, a mutant that produces an unusually long distal rod, and a homology model of FlgG constructed based on the crystal structure of St-FlgE [22]. The helical symmetry of the distal rod is almost the same as that of the hook. Therefore, the subunit arrangement of the rod is very similar to that of the hook, but the orientation of each domain is significantly different. The D1 domain of FlgG stands upright to tightly interact with the neighboring subunits in the protofilament of the distal rod, whereas that of FlgE is tilted about 7° to make a gap along the protofilament. Moreover, the N-terminal helix of FlgG is longer than that of FlgE, so the N-terminal helix directly contacts with the axially neighboring subunit in the rod. These tight axial subunit interactions are thought to provide the bending rigidity to the distal rod. However, both structural models do not include the Dc region due to the limited resolution of the density map. The Dc region contains a FlgG specific segment not present in FlgE, and this segment is important for the rod to form a rigid, straight structure. A FlgE mutant with the insertion of the FlgG specific segment forms a straight and rigid hook [23]. Therefore, a complete and more precise structural model of the rod including the Dc region is needed for further understanding of the molecular basis of its mechanical and functional properties.

Here, we have constructed an atomic model of the St-rod structure on the basis of the crystal structure of a core fragment of St-FlgG (FlgG20) solved at 2.0 Å resolution in combination with the previous cryoEM map. FlgG20 more closely resembles Cj-FlgE than the core fragment of St-FlgE. Cj-FlgE also contains the FlgG specific segment that is missing in St-FlgE. Therefore, the complete distal rod model was constructed by using the similarities between FlgG20 and Cj-FlgE. This model revealed that the Dc region adopts the l-stretch structure and that the intersubunit interactions mediated by the l-stretch greatly contribute to stabilizing and rigidifying the distal rod structure.

## 2. Materials and Methods

### 2.1. Preparation and Crystallization of FlgG20

Details of expression, purification, and crystallization of FlgG20 (residues 47–227) from *Salmonella enterica* serovar Typhimurium and its selenomethionine derivative have been previously reported [24]. Key resources are summarized in Table S1. Briefly, FlgG20 was purified by affinity chromatography using a HisPrep FF 16/10 column (GE Healthcare Life Sciences, Little Chalfont, Buckinghamshire, UK) and treated with thrombin-protease (GE Healthcare Life Sciences) to remove His-tag, followed by anion-exchange chromatography using a RESOURCE Q (6 mL) (GE Healthcare Life Sciences). The best crystals were obtained from sitting drops with a reservoir solution containing polyethylene glycol monomethyl ether 2000 (Hampton Research, Aliso Viejo, CA, USA), ammonium sulfate (WAKO) and sodium acetate (pH 4.6) (Hampton Research).

### 2.2. Diffraction Data Collection and Structure Determination

X-ray diffraction data were collected at synchrotron beamlines BL38B1 and BL41XU in SPring-8 (Harima, Japan) with the approval of the Japan Synchrotron Radiation Research Institute (JASRI) (Proposal No. 2010B1013 and 2010B1901), as previously described [24]. The diffraction data were processed using iMOSFLM [25] and were scaled using SCALA [26] from the CCP4 program suite [27,28]. Data collection statistics are summarized in Table 1. The initial phase was determined using the SAD data of the Se-Met derivative and the initial model was automatically constructed with a program Phenix [29]. The model was manually modified with Coot [30] and refined with Phenix. The refinement converged to an R value of 20.0% and an R free value of 22.9% for the SAD data at a resolution of 2.0 Å. The final model contains 304 residues and 181 water molecules. A Ramachandran plot showed 98.0% and 2.0% residues located in the most favorable and allowed regions, respectively. Refinement statistics are summarized in Table 1.

### 2.3. Model Building of the Rod and Hook

The crystal structure of FlgG20 is roughly fitted in the EM density map of the rod (EMDataBank ID: EMD-6683) using UCSF Chimera [31]. The D0–D1 region of Cj-FlgE (PDB ID: 5JXL) was superimposed on FlgG20 by fitting the D1 domains of both molecules. Then, D0 and the l-stretch of Cj-FlgE were added to FlgG20 and replaced their sidechains with those of St-FlgG to construct the full-length FlgG model. The model was modified by fitting in the EM density using Coot. The residues 53–64 were removed due to the poor density of this region. Then, a rod segment model composed of 22 subunits (four turns along the 1-start helix) was produced using the helical symmetry of the rod (1-start: *θ* = 64.75˚, *z* = 4.13 Å) and was refined with real space refinement using Phenix under noncrystallographic symmetry (NCS) constraints. The NCS operator was not refined to preserve helical symmetry.

The hook model was constructed basically the same way as the rod model. The D0–D1 region of Cj-FlgE (PDB ID: 5JXL) was superimposed on FlgE (PDB ID: 3A69) by fitting the D1 domains of both molecules. D0 and the l-stretch of Cj-FlgE were fused to the D1–D2 domain of St-FlgE and replaced their sidechains with those of St-FlgE. The model was modified by fitting in the EM density of the hook (EMDataBank ID: EMD-1647) using Coot. A rod segment model composed of 22 subunits (four turns along the 1-start helix) was produced using the helical symmetry of St-hook (1-start: *θ* = 64.78˚, *z* = 4.12 Å) and was refined with real space refinement under NCS constraints using Phenix. The refinement statistics are summarized in Table 1.

Coordinates and structure factors have been deposited in the Protein Data Bank under accession codes 6JF2 (FlgG20), 6JZR (St-rod) and 6JZT (St-hook).

## 3. Results

### 3.1. Structure of FlgG20

**Figure 1:**
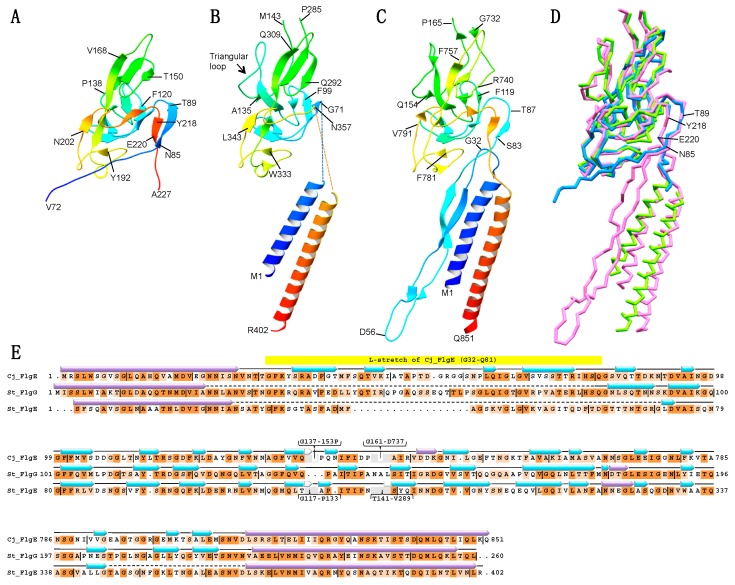
Structure of a core fragment of St-FlgG (FlgG20) from *Salmonella typhimurium* (St), FlgE from *Salmonella typhimurium* and FlgE from *Campylobacter jejuni* (Cj). Ribbon representation of the crystal structure of FlgG20 (**A**), the cryoEM structure model of D0 and D1 domains of St-FlgE (PDB ID: 3A69) (**B**), and the cryoEM structure of D0, l-stretch and D1 of Cj-FlgE (PDB ID: 5JXL) (**C**). The Dc region of the St-FlgE structure is not modeled due to the low resolution of the cryoEM map in (**B**). The models are color-coded from blue to red through the rainbow spectrum from the N to C terminus. (**D**) The superimposition of backbone models of FlgG20 (blue), St-FlgE (green) and Cj-FlgE (pink). The positions of N85, T89, Y218 and T221 of FlgG20 are indicated. (**E**) Structure-based sequence alignment of Cj-FlgG, St-FlgG and St-FlgE. Conserved residues are highlighted in dark orange (identical residues) or light orange (similar residues). Purple and cyan arrow indicates α-helix and β-strand, respectively. Molecular figures were drawn using MolFeat (Ver 3.6, FiatLux Corporation).

The crystal asymmetric unit contains two FlgG20 molecules, mol A (80–224) and mol B (72–227), whose structures are almost identical to each other with a root-mean-square deviation (rmsd) of 0.60 Å for Cα atoms. The N-terminal 33 and C-terminal 3 residues of mol A and 25 residues of mol B were not traced because of poor electron density. FlgG20 shows a single domain structure composed of 15 β-strands and the loops connecting the β-strands (Figure 1A), and is similar to the D1 domain of FlgE except for the absence of the triangular loop (Figure S1A,B) and the conformation of the N- and C-terminal regions (Figure 1B). Interestingly, the N- and C-terminal regions of FlgG20 are more similar to *Campylobacter* FlgE (Cj-FlgE, PDB ID: 5JXL) [15] than the core fragment of *Salmonella* FlgE (St-FlgE31, PDB ID: 1WLG) [18] (Figure 1 and Figure S1). The β-stretch composed of the N- and C- terminal chains of FlgG20 (N85–T89 and Y218–T221, respectively) fits nicely on the corresponding regions of Cj-FlgE (Figure 1D).

### 3.2. Atomic Model of the Distal Rod

The previous atomic model of the distal rod (PDB ID: 5WRH) was built by fitting a homology model of a fragment of FlgG (92–216) into a cryoEM map of the polyrod [22]. Although the homology model is similar to the D1 core of the FlgG20 crystal structure, the crystal structure includes a part of the region connecting D0 and D1 that was not present in the homology model (Figure S1). We, therefore, conducted rigid body fitting of the FlgG20 structure into the cryoEM map (EMDataBank ID: EMD-6683) using UCSF Chimera, and compared it with the previous model [22]. The C-terminal region of FlgG20 comes close to the N-terminal end of the C-terminal D0 helix of FlgG in the previous rod model. The N-terminal region of FlgG20 goes into the edge of the elongated density that was not assigned in the previous model. We found here that the elongated density can be assigned as the l-stretch, as is seen in Cj-FlgE, considering the structural similarity between FlgG20 (Figure 1A) and Cj-FlgE (Figure 1C) and the amino acid sequence similarity between St-FlgG and Cj-FlgE (Figure 1E). We superimposed the D0-l-stretch-D1 structure model of Cj-FlgE on the FlgG20 model fitted in the cryoEM map and realized that it is possible to fit the D0-l-stretch structure of Cj-FlgE into the elongated density with slight modification. Then, we constructed a new subunit model of the distal rod by connecting the FlgG20 model with the D0-l-stretch model built on the basis of the Cj-FlgE structure.

**Figure 2:**
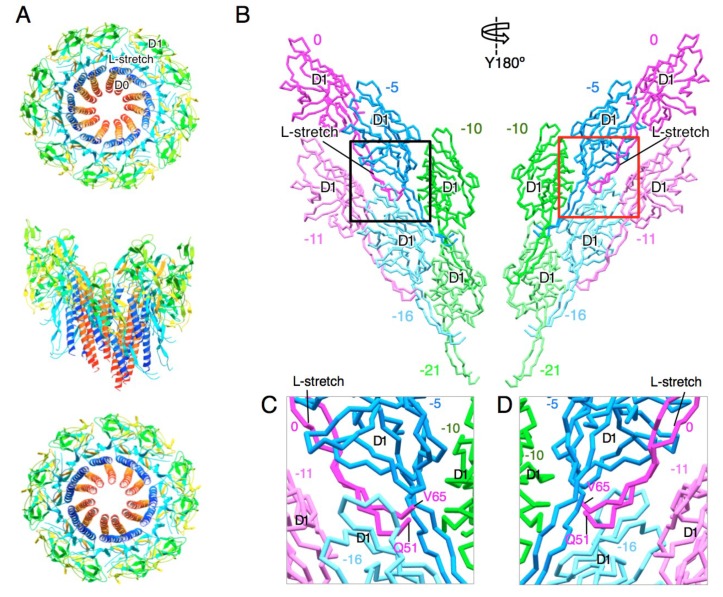
Structure model of the rod. (**A**) Two turns of the distal rod model. Eleven FlgG subunits along the one start helix are shown. Each subunit is colored from blue to red through the rainbow spectrum from the N to C terminus. Upper panel, viewed from the distal end; middle panel, side view; lower panel, viewed from the proximal end. (**B**) Subunit interaction of the D1 domain and the L-stretch in the rod. Six subunits in three adjacent protofilaments (two subunits from each protofilament) are shown. The number counted from the subunit 0 along the 1-start helical line is shown above or below each subunit. Left panel, view from the outside; right panel, view from the inside of the rod. (**C**) (**D**) Close up view of the L-stretch of the rod. The region in the black and red boxes in (**B**) are shown in (**C**) and (**D**), respectively. The tip of the L-stretch (P52-S64) is invisible due to poor density. Molecular figures were drawn using MolFeat (Ver 3.6, FiatLux Corporation).

The new model revealed that the l-stretch extensively contacts with the D1 domains of other FlgG subunits and thereby reinforces the rod structure (Figure 2). The l-stretch extends along the D1 domain of FlgG in the −5 position toward that in the −10 position (Figure 2B–D). The location of the tip of the l-stretch (P52–S64) is unclear due to the poor density of this region. The l-stretch also interacts with the D1 domains of FlgG in the −11 and −16 positions and thereby mediates the 6-start interactions of the subunits in the −5 and −11 positions and those in the −10 and −16 positions (Figure 2C,D). The D1 domain interacts with the D1 domains of nearest-neighbor subunits in all directions, but only a few direct contacts are observed in each direction (Figure S2). Thus, the interactions mediated by the l-stretch greatly contribute to stabilizing and rigidifying the distal rod.

### 3.3. Atomic Model of the Hook

**Figure 3:**
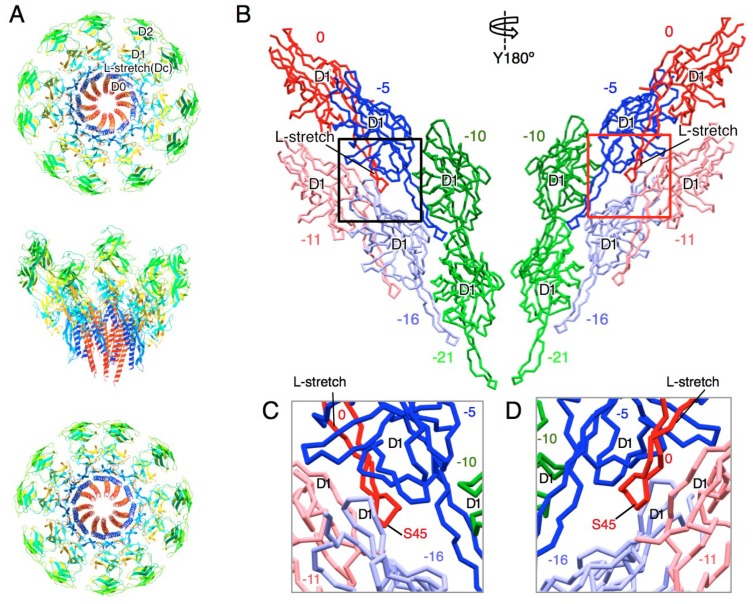
Structure model of the hook. (**A**) Two turns of the hook model. Eleven FlgE subunits along the one start helix are shown. Each subunit is colored from blue to red through the rainbow spectrum from the N to C terminus. Upper panel, viewed from the distal end; middle panel, side view; lower panel, viewed from the proximal end. (**B**) Subunit interaction of the D1 and the l-stretch in the hook. Six subunits in three adjacent protofilaments (two subunits from each protofilament) are shown. The number counted from the subunit 0 along the 1-start helical line is shown above or below each subunit. Left panel, view from the outside; right panel, view from the inside of the hook. (**C**) (**D**) Close up view of the L-stretch in the hook. The region in the black and red boxes in (**B**) are shown in (**C**) and (**D**), respectively. Molecular figures were drawn using MolFeat (Ver 3.6, FiatLux Corporation).

The previous cryoEM map of the St-hook also showed an unassigned elongated density between the D1 domain and the D0 helices [21]. The shape and position of the density is very similar to the density corresponding to the l-stretch of the Cj-hook or that of the St-rod. Therefore, we superimposed the D0-l-stretch-D1 structure of Cj-FlgE (PDB ID: 5JXL) on the St-FlgE model (PDB ID: 3A69) in the cryoEM density of the St-hook (EMDataBank ID: EMD-1647). The D0 helices and the l-stretch of Cj-FlgE were well fitted into the EM density with slight modification (Figure 3). The amino acid sequence alignment indicates that the l-stretch of St-FlgE is 18 residues shorter than that of Cj-FlgE (Figure 1E) [32]. Consistent with this, the density of St-FlgE corresponding to the l-stretch is shorter than the other two and terminates at the position expected from the sequence. Thus, we built the model of the Dc region based on the Cj-FlgE structure and constructed a full-length St-hook model. The subunit interaction mediated by the l-stretch is not as extensive as in the distal rod (Figure 2 and Figure 3). The l-stretch extends to the −5 direction and interacts with the inner surface of the D1 domains of the FlgE subunits in the −5 and −11 positions but not with the subunits in −10 and −16 positions (Figure 3B–D).

## 4. Discussion

The distal rod and hook show distinct mechanical properties, although they share the same helical symmetry and repeat distance and are composed of similar subunit proteins (FlgG and FlgE) with 39% sequence identity. The rod is straight and rigid whereas the hook is curved and flexible and is easy to bend. Previous cryoEM studies revealed that the D1 domain of FlgE in the hook is tilted about 7° relative to that of FlgG in the distal rod. this difference resulted in a loose axial subunit packing of FlgE, making the hook flexible in bending unlike the rod. Moreover, a small gap between the D0 helices of the axially contacting FlgE subunits allows compression and extension of each protofilament [22].

The new models of the rod and hook have revealed marked contributions of the l-stretch to their mechanical properties. Interacting with the −5 and −10 subunits, the long l-stretch of FlgG fastens three adjacent protofilaments in the rod. In addition, the l-stretch mediates the 6-start interaction of −5 and −11 subunits and 0 and −11 subunits. These 5-start and 6-start interaction networks make the distal rod rigid in its straight form. Moreover, the tight interaction of l-stretch with the D1 domain in the −5 position may stabilize the upright orientation of FlgG, forming tight subunit packing in the distal rod. In contrast, the l-stretch of FlgE is shorter than that of FlgG and only interacts with the inner surface of the −5 and −10 subunits. These interactions probably reinforce the hook structure while allowing the axial compression and extension of each hook protofilament for the bending flexibility of the hook.

**Figure 4:**
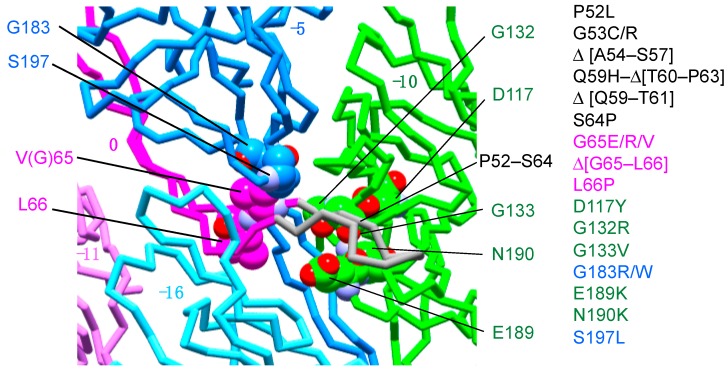
Polyrod mutation sites are localized around the tip of the l-stretch. The known polyrod mutation sites are mapped on the rod structure. Subunit at 0, −5, −10 positions are colored in magenta, blue, and green, respectively. The putative structure of the l-stretch tip of subunit 0 is shown in gray. The polyrod mutation sites except for those in the l-stretch tip are indicated by ball models. Oxygen and nitrogen atoms are colored in red and purple, respectively. The carbon atoms are painted by the same color as used for the subunits. The polyrod mutations [32] are listed to the right of the figure. Molecular figures were drawn using MolFeat (Ver 3.6, FiatLux Corporation).

The rod length is regulated to around 25 nm in wild-type cells, but some FlgG mutants form unusually long rod structures, termed polyrods. More than 20 mutants, including a few residue deletion mutants, were isolated, and over half of the mutation sites were localized in the segment of residues 52–66 [32,33,34], which corresponds to the distal part of the l-stretch. Chevance et al. mapped the mutation sites on a homology model of FlgG and suggested that this region might interact with residues G183 and S197 in the neighboring FlgG subunit in the rod and that the other mutation sites are rather widely distributed [32]. We have now mapped these mutation sites on the new rod model and found that all of them are localized within the plausible space where the distal part (residues 52–66) of the l-stretch would be located, albeit most of them were not visible in the EM map (Figure 4). The absence of the EM density for the distal part of the l-stretch may be because the resolution of the EM density map was not high enough to clearly resolve this part, which is in close contact with neighboring subunits, as described above. The distal part of the L-stretch in the 0 position presumably interacts with the middle part of the l-stretch of the subunit in the −5 position and the D1 domain of the subunit in the −10 position. Therefore, the intermolecular interactions around the distal part of the l-stretch are likely to be the key factor for the stiffness of the rod. To confirm this idea, we need a cryoEM structure of the polyrod mutant, as well as a wild-type distal rod at a higher, near-atomic resolution.

## 5. Conclusions

We constructed a structural model of the flagellar distal rod by fitting the crystal structure of FlgG20 into the previous cryoEM map of a polyrod and a new hook model using the previous cryoEM map of the hook. These structural models suggest that the l-stretch stabilizes the distal rod and plays a key role in achieving the distinct mechanical properties of the rod using a structurally similar protein to that of the hook.

## Figures and Tables

**Table 1:** Data collection and refinement statistics.

Space Group	*P*2_1_2_1_2_1_		
Cell dimensions (Å)	a = 47.5, b = 67.0, c = 110.3		
Wavelength (Å)	0.9790		
Resolution (Å)	36.8–2.0 (2.11–2.00)		
R*_merge_*	0.106 (0.389)		
*I/σI*	10.8 (4.0)		
Completeness (%)	98.1 (97.5)		
Redundancy	6.9 (6.6)		
	FlgG20	Rod (EMD-6683)	Hook (EMD-1647)
Resolution range (Å)	36.6–2.0 (2.08–2.00)	7.4	7.1
No. of reflections working	22,747 (2448)		
No. of reflections test	1217 (124)		
*R_w_* (%)	20.0 (24.8)		
*R_free_* (%)	22.9 (31.8)		
No. of protein atoms	2264		
No. of solvent atoms	192		
B-factors			
Protein atoms	37.0		
Solvent atoms	38.5		
Map correlation coefficient		0.729	0.713
Root mean square deviation bond length (Å)	0.002	0.03	0.00
Root mean square deviation bond angle (°)	0.537	0.73	0.59
Ramachandran plot (%)			
favored	98.0	93.0	94.3
allowed	2.0	7.0	5.7
outliers	0	0	0
Rotamer outliers (%)	0.4	9.27	7.14
All atom clash score		7.99	4.80

Values in parentheses are for the highest resolution shell. R_w_ = ∑ || Fo | − | Fc ||/∑ | Fo |, R_free_ = ∑ || Fo | − | Fc ||/∑ | Fo |.

## Supplementary Materials

The following are available online, Figure S1: Structural comparison of the D1 domain, Figure S2: The rod model docked in the cryoEM map, Table S1: Key Resources.
