# Supplementary material for: Architecture of the Bacterial Flagellar Distal Rod and Hook of Salmonella
Source: Biomolecules. 2019 Jul 7;9(7):260. doi: 10.3390/biom9070260 (PMC6681337; doi:10.3390/biom9070260)
Supplement: Supplementary file 1 [file biomolecules-09-00260-s001.pdf]

biomolecules

MDPI

Table S1. Key Resources

REAGENT or RESOURCE	*SOURCE*	*IDENTIFIER*
Chemicals and Protease		
Ammonium sulfate	WAKO	019-03435
PEG MME 2000	Hampton Research	HR2-613
Sodium acetate trihydrate pH 4.6, 1.0 M solution	Hampton Research	HR2-731
Thrombin	GE Healthcare Life Sciences	27084601
Columns		
HisPrep FF 16/10 column	GE Healthcare Life Sciences	28936551
RESOURCE Q (6 ml)	GE Healthcare Life Sciences	17117901
Deposited Data		
Amino acid sequence of FliC	Uniprot https://www.uniprot.org/	UniProtKB ID: P06179
Amino acid sequence of FlgE	Uniprot https://www.uniprot.org/	UniProtKB ID: P0A1J1
Amino acid sequence of FlgG	Uniprot https://www.uniprot.org/	UniProtKB ID: P0A1J3
Structure of FlgG20	This study	PDB ID: 6JF2
Structure of the polyrod from *Salmonella typhimurium*	This study	PDB ID: 6JZR
Structure of the hook from *Salmonella typhimurium*	This study	PDB ID: 6JZT
Structure of the hook from *Salmonella typhimurium*	Fujii et al., 2009	PDB ID: 3A69<br>EMDataBank ID: EMD-1647
Structure of the polyrod from *Salmonella typhimurium*	Fujii et al., 2017	PDB: 5WRH<br>EMDataBank ID: EMD-6683
Atomic coordinates from *Campylobacter jejuni*	Matsunami et al., 2016	PDB ID: 5JXL
Software and Algorithms		
CCP4 program suite	Collaborative Computational Project Number 4, 1994; Winn et al., 2011	http://www.ccp4.ac.uk/
UCSF Chimera	Pettersen et al., 2004	https://www.cgl.ucsf.edu/chimera/
COOT	Emsley et al., 2010	https://www2.mrc-lmb.cam.ac.uk/personal/pemsley/coot/
iMOSFLM	Battye et al., 2011	https://www.mrc-lmb.cam.ac.uk/harry/imosflm/ver722/introduction.html
MolProbity	Chen et al., 2010	http://molprobity.biochem.duke.edu/
Phenix	Adams et al., 2010	https://www.phenix-online.org/
SCALA	Evans, 2006	https://www.mrc-lmb.cam.ac.uk/harry/pre/scala.html
MolFeat Ver 3.6	FiatLux Corporation	https://www.fiatlux.co.jp/link.html

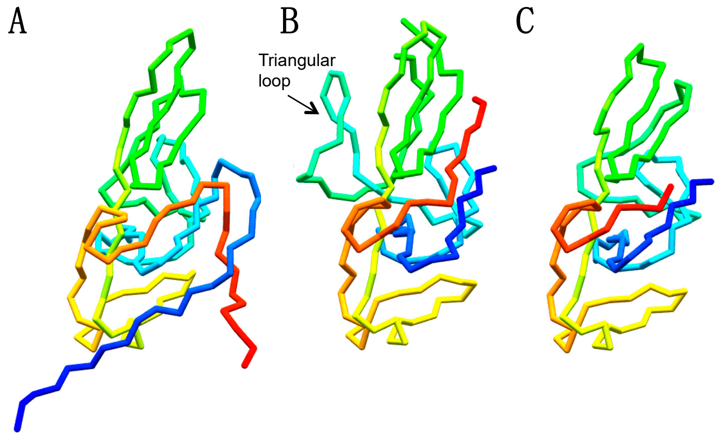

Figure S1. Structural comparison of the D1 domain. (**A**) FlgG20, (**B**) St-FlgE (PDB ID: 1WLG), and (**C**) the homology model of FlgG (PDB ID: 5WRH) are shown in Cα trace representation. Molecular figures were drawn using MolFeat (Ver 3.6, FiatLux Corporation).

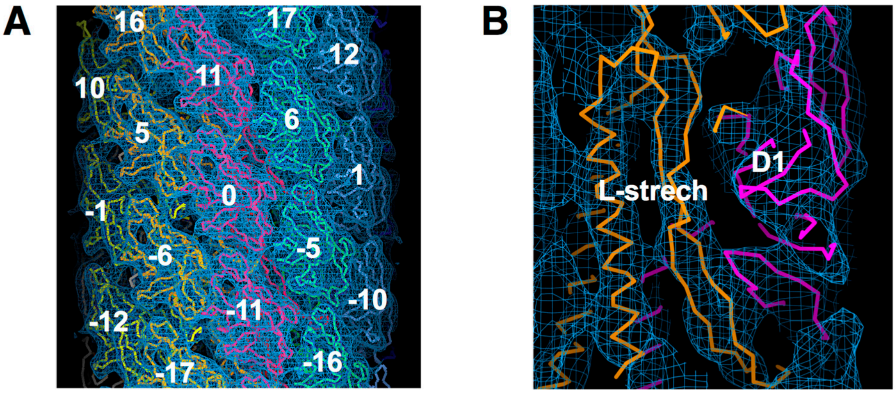

Figure S2. The rod model docked in the cryoEM map. (**A**) Subunit packing of FlgG in the rod. The Cα trace models are fitted in the density map. The subunits surrounding subunit 0 are labeled with the number showing the direction of the helical line. (**B**) Close up view of the L-stretch of the rod. The figures were drawn using CCP4MG (Ver.2.10.10) [35].

35. McNicholas, S.; Potterton, E.; Wilson, K.S.; Noble, M.E.M. Presenting your structures: the CCP4mg molecular-graphics software. *Acta Crystallogr. D Biol. Crystallogr.* **2011**, *67*, 386–394, doi:10.1107/S0907444911007281.
